# Innate Immune Response to Rift Valley Fever Virus in Goats

**DOI:** 10.1371/journal.pntd.0001623

**Published:** 2012-04-24

**Authors:** Charles K. Nfon, Peter Marszal, Shunzhen Zhang, Hana M. Weingartl

**Affiliations:** 1 National Center for Foreign Animal Disease, Canadian Food Inspection Agency, Winnipeg, Manitoba, Canada; 2 Department of Microbiology, University of Manitoba, Winnipeg, Manitoba, Canada; University of Texas Medical Branch, United States of America

## Abstract

Rift Valley fever (RVF), a re-emerging mosquito-borne disease of ruminants and man, was endemic in Africa but spread to Saudi Arabia and Yemen, meaning it could spread even further. Little is known about innate and cell-mediated immunity to RVF virus (RVFV) in ruminants, which is knowledge required for adequate vaccine trials. We therefore studied these aspects in experimentally infected goats. We also compared RVFV grown in an insect cell-line and that grown in a mammalian cell-line for differences in the course of infection. Goats developed viremia one day post infection (DPI), which lasted three to four days and some goats had transient fever coinciding with peak viremia. Up to 4% of peripheral blood mononuclear cells (PBMCs) were positive for RVFV. Monocytes and dendritic cells in PBMCs declined possibly from being directly infected with virus as suggested by *in vitro* exposure. Infected goats produced serum IFN-γ, IL-12 and other proinflammatory cytokines but not IFN-α. Despite the lack of IFN-α, innate immunity via the IL-12 to IFN-γ circuit possibly contributed to early protection against RVFV since neutralising antibodies were detected after viremia had cleared. The course of infection with insect cell-derived RVFV (IN-RVFV) appeared to be different from mammalian cell-derived RVFV (MAM-RVFV), with the former attaining peak viremia faster, inducing fever and profoundly affecting specific immune cell subpopulations. This indicated possible differences in infections of ruminants acquired from mosquito bites relative to those due to contact with infectious material from other animals. These differences need to be considered when testing RVF vaccines in laboratory settings.

## Author Summary

Rift Valley fever (RVF) is a mosquito-transmitted disease of ruminants and man, which occurs in Africa, Saudi Arabia and Yemen but could spread to other areas. There isn't much information on some aspects of the immune response to this disease and how it affects cells of the immune system in the natural animal hosts. To fill in some of this knowledge gap, we studied RVF in goats experimentally infected with the RVF virus. We also compared RVF virus grown in an insect cell-line and that grown in a mammalian cell-line for differences in the course of infection. Virus was present in the blood of the goats one day after infection. Some goats had fever coinciding with the time when the virus level in the blood was highest. Some cells in the blood dropped in number possibly as a direct effect of virus. Infected goats secreted cytokines (interferon gamma and interleukin-12), which possibly contributed to protection against RVF. Virus from an insect cell-line appeared to have more obvious effects in infected goats suggesting that differences may exist in infections of ruminants acquired from mosquito bites compared to those due to contact with infectious material from other animals.

## Introduction

Rift Valley fever (RVF) is a disease of ruminants and man caused by the mosquito transmitted Rift Valley fever virus (RVFV), genus *Phlebovirus*, family *Bunyaviridae* [1]. This spherical shaped, enveloped virus has a negative-sense single-stranded RNA genome made up of 3 segments. The large (L) segment encodes for the viral RNA-dependent RNA polymerase while the medium (M) segment encodes the external glycoproteins (Gn and Gc) and the non-structural protein (NSm). The small (S) segment is ambisense, coding for the nucleoprotein (N) in the antigenomic sense and the non-structural protein (NSs) in the genomic direction [2].

RVF outbreaks are frequently reported in Sub-Saharan African countries where the disease is endemic. These include Kenya, Tanzania, Somalia, South Africa, Sudan, Uganda, Madagascar and Senegal. However, outbreaks were also reported in Egypt, Yemen and Saudi Arabia indicating an expanding range for this disease [3]. RVFV is transmitted primarily by *Aedes* and *Culex* mosquitoes, with the latter serving as a magnifying host during outbreaks [2] . In addition to infectious mosquito bites, humans can also acquire RVF through contact with blood of diseased animals [4], [5]. Outbreaks of RVF in endemic countries usually coincide with conditions such as periods of heavy rainfall and flooding, which favour heavy breeding of mosquito vectors [6], [7].

RVF is characterized by large abortion storms and close to 100% mortality in newborn sheep, goats and cattle resulting in severe adverse socio-economic effects [8]. These animals carry high titres of virus (6 log_10_ to 8 log_10_ PFU/mL) in their blood resulting in fever, inappetence, nasal discharges and diarrhoea [3]. However, adult sheep, goats and cattle are more resistant to RVFV and experience lower mortality rates between 10–30% [3]. Human RVF usually manifests as a mild and self-limiting fever, but in some patients may progress to a haemorrhagic fever, neurological disorder or blindness [2], [3].

Innate and adaptive immune responses contribute to the clearance of RVFV in infected animals [3], [9]. Evidence for the role of innate immunity is mostly based on results from experimental models [9]–[12]. Interferon alpha (IFN-α) is believed to protect against RVFV because monkeys that secreted this cytokine within 12 h of being challenged with RVFV did not develop disease [11]. However, RVFV NSs protein inhibits IFN-α and IFN-β production/induction, thereby enabling early replication and viremia [12]–[14]. Anti-RVFV antibodies are detectable 4 to 8 days following infection [15]–[17]. Neutralising antibodies are believed to be crucial for the protection of infected animals [2], [11].

Although ruminants have since been recognized as the primary animal hosts, there is little knowledge of the pathogenesis of RVFV in goats. In 2–3 months old goats experimentally infected with RVFV, viremia was detected 24 h post subcutaneous inoculation and lasted for 3 days [18]. These goats also had a mild transient increase in rectal temperature. Mild fever was equally observed in goats inoculated by inhalation and virus could be recovered from throat washes 2 days after inoculation [18]. In addition, virus was apparently transmitted to contact goats. Clinical signs varied in severity depending on the route of inoculation and included lethargy, diarrhoea and occlusion of the eyes. All the goats died between days 9 and 70 post inoculation possibly due to RVF but secondary infections could have also contributed to these deaths. Gross and histopathology lesions were observed in the liver, lungs, kidneys, spleen and brain of infected goats [18]. An attenuated live RVFV vaccine (Smithburn strain) has also been shown to cause abortion in vaccinated pregnant goats and pathology in the liver, kidney and other organs of vaccinated kids [19].

There is still a remarkable paucity of data on RVFV innate and cell mediated immune responses in sheep, goats and cattle. Knowledge of the pathogenesis and immune response to RVFV in these domestic ruminants is crucial for rational design of new vaccines and/or evaluation of existing vaccines for veterinary and human use. Therefore, to better understand RVF in small ruminants, we performed experimental infection of goats with RVFV.

The C-type lectins, DC-SIGN and L-SIGN have been identified as probably receptors for arthropod borne viruses (arboviruses) [20]. Similarly, DC-SIGN has recently been identified as a receptor for *Phleboviruses* including RVFV [21]. Furthermore, insect cell-derived arboviruses belonging to the *Alphavirus* genus were more infectious to monocyte-derived dendritic cells (MoDCs) compared to mammalian cell-derived virus [20], [22] possibly due to their stronger recognition and binding to the C-type lectin receptors. In addition, insect cell-derived *Alphavirus* was poor at inducing type 1 interferon responses in MoDCs, further enhancing its ability to replicate in these cells [22]. These data collectively suggest that there might be differences between insect cell-derived and mammalian cell-derived arbovirus in *in vivo* infectivity and disease pathogenesis in susceptible animals. To investigate this, we inoculated goats with insect cell-derived and mammalian cell-derived RVFV and monitored *in vivo* differences in the course of infection. In addition, we evaluated RVFV from these 2 sources for differences in *in vitro* infectivity of MoDCs.

## Materials and Methods

### Ethics statement

All animal experiments were carried out in enhanced biosafety level 3 (BSL3+) at the National Centre for Foreign Animal Disease (NCFAD) Winnipeg, Manitoba. All protocols for animal use, under animal use document (AUD) number C-09-004, were approved by the Canadian Science Center for Human and Animal Health, Winnipeg, Manitoba, Canada Animal Care Committee. Only the NCFAD veterinarian and trained animal care personnel were allowed access to the animals. Care was taken to minimise animal suffering, respecting the Canadian Council on Animal Care guidelines for animal manipulations.

### Virus production and titration

RVFV strain ZH501 [23] was kindly provided by Dr Heinz Feldmann, National Microbiology Laboratory, Winnipeg, Canada.

The mammalian cell-derived RVF virus (MAM-RVFV) was propagated on Vero E6 cells (American Tissue Culture Collection, ATCC, Manassas, VA, USA). Infection of Vero E6 cells with RVFV was done in dulbecco's modification eagle's medium (DMEM) supplemented with 0.3% bovine serum albumin (BSA, Wisent, QC, Canada) at an MOI of 0.1 and the cultures maintained in DMEM with 0.3% BSA at 37°C, 5% CO_2_ and 95% relative humidity. The virus eventually used as inoculum for goats was from the 4^th^ passage in Vero E6 cells.

Insect cell-derived RVFV (IN-RVFV) was obtained by propagating the passage 3 RVFV from Vero E6 cells above in a mosquito cell line (C6/36, ATCC). C6/36 cells were infected at an MOI of 0.1 and maintained at 28°C in a 1∶1 mixture of EMEM (Wisent) and ESF-921 (Expression Systems, Woodland, CA, USA) supplemented with 2.5% FBS, 25 mM HEPES and 1 mM sodium pyruvate. The IN-RVFV eventually used as inoculum for goats was from the 2^nd^ passage in C6/36 cells.

The sequences for the M and S segments of MAM-RVFV and IN-RVFV were compared for any differences that might result from propagation in the different cell lines. The M segment was selected for sequencing because it was recently shown that a single nucleotide substitution in the glycoprotein (Gn) can have a significant effect on the virulence of RVFV [24]. In addition, the NSs protein encoded by the S segment is also of importance in the virulence of RVFV [13], [14], [25]. For sequencing, viral RNA was isolated as previously described [26] and RT-PCR performed using published primers and protocol [27]. The RT-PCR products were purified and then cloned using the cloneJet2.1/blunt vector and the CloneJet PCR cloning kit (Fermentas, Canada). Three positive clones per gene segment were identified and sequenced and a consensus sequence obtained as previously described [28].

Vero E6 cells were used to determine the titres of RVFV derived from both cell lines. Briefly, serial 10 fold dilutions of virus in 200 µL DMEM were transferred onto a 24-well plate containing confluent Vero E6 cell monolayer. After 1 h, at 37°C, 5% CO_2_ and 95% relative humidity, an overlay of 1.75% carboxymethylcellulose in DMEM containing 0.3% BSA (CMC overlay) was added to all wells and plates incubated as above. After 4–5 days cells were fixed with 10% formalin, stained with 0.5% crystal violet and plaques counted.

### Goat inoculation

Healthy 4 month old Boer-cross goats were obtained from breeders in Manitoba, Canada and allowed 10 days to acclimatize to BSL3+ containment at NCFAD, during which they were monitored daily for any signs of disease. After acclimatization, the goats were divided into 2 groups (4 per group) and housed in separate cubicles. One group was inoculated with 5 log_10_ PFU of IN-RVFV and the 2^nd^ group with 5 log_10_ PFU of MAM-RVFV per animal by the subcutaneous route. Daily monitoring was continued and rectal temperatures recorded. Blood for serum samples and for peripheral blood mononuclear cells (PBMCs) isolation was collected prior to and daily for the first 7 days post infection with RVFV. Additional sampling was done at 14, 21 and 30 DPI. Serum samples were stored at −70°C.

### Peripheral blood mononuclear cells (PBMCs) isolation and flow cytometry

Blood for PBMCs isolation was collected in EDTA-treated vacutainers prior to and daily for the first 7 days post infection (DPI) of goats with RVFV. PBMCs were purified from this blood using Ficoll-Paque Plus (GE Healthcare Bio-Sciences AB, Uppsala, Sweden) with minor modifications to manufacturer's protocol. Briefly, blood was mixed with an equal volume of sterile phosphate buffered saline (PBS, pH 7.2, Sigma), layered over Ficoll-Paque Plus and centrifuge at 800 g for 30 min with the centrifuge brake off. The PBMC layer was collected and washed twice with PBS.

The cells were then resuspended in FACS buffer (PBS containing 0.1% BSA and 0.1% sodium azide) and stained for flow cytometry using antibodies known to cross react with goat cell surface markers [29]. Approximately 10^6^ PBMCs/tube were each stained with mouse anti bovine CD5:FITC (clone CC17), mouse anti sheep CD8:RPE (clone 38.65), mouse anti bovine CD21:RPE (cloneCC21) all from AbD Serotec (Oxford, UK) or mouse anti CD172a (SWC3, clone DH59B) from VMRD (Pullman, WA, USA) on ice for 30 min. Isotype control antibodies were included to check for non-specific binding. Cells were washed twice with FACS buffer and for CD172a (unlabelled primary antibody), rat anti mouse IgG1:FITC (AbD Serotec) was added for another 30 min on ice.

For RVFV detection, PBMCs from infected goats were permeabilized using BD cytofix:cytoperm reagent (BD Biosciences, San Diego, CA, USA) according to manufacturer's protocol. An optimal amount of rabbit polyclonal anti RVFV NSm1 antibody was then added to the cells and incubated on ice for 30 min. The rabbit polyclonal anti RVFV NSm1 antibody (R1108) was produced by the EvoQuest Team, Invitrogen (Carlsbad, California, USA) using a synthetic NSm1 polypeptide. Antibody from a naïve rabbit was used as isotype control. Cells were washed twice with BD perm/wash buffer, then stained with Alexa Fluor 594 donkey anti rabbit IgG (Invitrogen, Oregun, USA) for 30 min on ice, followed by 2 more washes with BD perm/wash buffer.

After the final wash in all staining protocols, cells were fixed overnight in 10% phosphate-buffered formalin before running on the FC500 two laser flow cytometer (Beckman Coulter). At least 25,000 events were acquired per sample and data analysed with the CXP analysis software (Beckman Coulter).

### Generation and infection of goat monocyte-derived dendritic cells

Peripheral blood mononuclear cells isolated from naïve goats as described above were resuspended in RPMI supplemented with 10% FBS, 100 U/mL penicillin, 100 µg/mL streptomycin, 200 µM/mL glutamax, 10 mM HEPES and 0.5 µM 2-mercaptoethanol (complete medium) and incubated in cell culture flasks at 37°C overnight for monocytes to attach. Non-adherent cells were removed, adherent monocytes washed twice with sterile PBS and then incubated at 37°C, 5% CO_2_ and 95% relative humidity in complete medium containing 1 in 10 dilution of recombinant bovine GM-CSF and 0.1 µg/mL recombinant bovine IL-4 (both from Serotec). These conditions have been shown to differentiate bovine monocytes into MoDCs [30]. As controls, adherent monocytes were also cultured in complete medium only (MΦ). Medium was supplemented after 3 days and MoDCs and MΦ harvested after 7 days. Flow cytometry for CD14, CD172a and CD11c surface markers was performed as described above.

For RVFV infection, approximately 5×10^5^ MoDCs were exposed to either IN-RVFV or MAM-RVFV at 0.1 MOI in RPMI without FBS for 1 h at 37°C, 5% CO_2_ and 95% relative humidity. The cells were then washed, resuspended in complete medium and incubated at 37°C for 24 h after which supernatants were harvested. The amount of virus in culture supernatants was measured by plaque assay on Vero E6 cells as described earlier.

### Determination of adaptive cell-mediated immunity to RVFV

Recall cell-mediated immunity (CMI) was determined by measuring RVFV-specific IFN-γ response [31] in PBMCs from goats at DPI 21. Antigen-specific induction of IFN-γ is one of the accepted methods in immunology for detecting CMI. PBMCs were isolated as described above and resuspended in complete medium. PBMCs were adjusted to 10^7^/mL, 100 µL added per well of a 96-well plate and duplicate wells stimulated with IN-RVFV or MAM-RVFV at 0.1 MOI to a final volume of 200 µL/well. Complete medium was added to negative control wells while ConA was used as positive control. Plates were incubated at 37°C, 5% CO_2_ and 95% relative humidity for 48 h, supernatants harvested and stored at −70°C for subsequent IFN-γ ELISA as described below.

### RNA extraction and qRT-PCR

RVFV RNA was extracted from serum using the TriPure Isolation reagent (Roche) according to the manufacturer's protocol. The purified RNA was stored at −70°C. Primers (Invitrogen) and probe (Applied Biosystems) designed to target nucleotides 2912 to 2981 of the RVFV L gene segment [26], [32] were used for quantitative real-time reverse transcriptase polymerase chain reaction (qRT-PCR) as previously described [26].

### Cytokine ELISA

Antibody pairs known to cross-react with goat IL-12 and IFN-γ were obtained from AbD Serotec and used as previously described [29]. Based on the cross-reactivity of other bovine and ovine antibodies with related targets in goats [29], [33], there was a high probability that other sheep and bovine cytokine ELISA antibodies will also cross-react with goat. We therefore obtained cytokine ELISA kits for sheep TNF-α, IL-6 and IL-1β from TSZ ELISA (Framingham, MA, USA) and bovine IFN-α from USCN Life Science Inc. (Wuhan, China) and used them with goat serum according to the manufacturers' instructions.

### Interferon gamma antiviral assay

To test the *in vitro* antiviral effect of ruminant IFN-γ against RVFV, recombinant bovine IFN-γ (RB- IFN-γ, Thermo Scientific) and Mardin-Darby bovine kidney (MDBK) cells were used. Approximately 2×10^5^ cells/well in 250 µL AMEM were added to a 24-well plate and an equal volume of various concentrations of RB- IFN-γ added in quadruplicates. Medium only was added to cells in the control wells. Plates were incubated at 37°C, 5% CO_2_ and 95% relative humidity for 24 h and checked for confluence. Well contents were emptied and 100 PFU of RVFV in 200 µL added to all wells except the cell controls. After 1 h at 37°C, CMC overlay was added to all wells and plates incubated at 37°C, 5% CO_2_ and 95% relative humidity. On day 3 after addition of virus, cells were fixed with 10% formalin and stained with 0.5% crystal violet. Plaques were counted in all wells and the percent plaque inhibition calculated.

### Antibody detection

Neutralising antibody response to RVFV was determined by plaque reduction neutralization test (PRNT) modified from a previously described protocol [34]. Serial 2-fold dilutions of serum in DMEM were made starting from 1 in 20 to obtain triplicates of 100 µL/well for each serum sample. 100 µL of DMEM containing 100 PFU of RVFV was added to each serum dilution, mixed and incubated at 37°C, 5% CO_2_ and 95% relative humidity for 1 h. 200 µL of the virus/serum mixture was then transferred onto a 24-well plate containing confluent Vero E6 cell monolayer and incubated for another 1 h. CMC overlay was then added to all wells and plates incubated at 37°C, 5% CO_2_ and 95% relative humidity. Assay of negative and positive control sera as well as a back titration of the virus was performed at the same time as the test sera. After 5 days the cells were fixed with 10% formalin, stained with 0.5% crystal violet and plaques counted. The reciprocal of the highest serum dilution that prevented at least 70% CPE was taken as the PRNT_70_ titre for that sample.

### Statistical analysis

Data from multiple time points was analyzed by ANOVA with the Dunnett multiple comparisons test using GraphPad InStat version 3.06 (GraphPad Software, San Diego, CA). Differences between groups for data collected at a single time point were analysed using the Student t-test. A p ≤ 0.05 was considered statistically significant.

## Results

### Sequence identity of the Rift Valley fever virus

The M gene sequences of MAM-RVFV and IN-RVFV were identical to each other (data not shown) as well as to a published sequence for the M segment (GenBank accession number DQ380200) of RVFV ZH501 strain [27]. Similarly, the gene sequences for the S segment of MAM-RVFV, IN-RVFV and a GenBank publication (accession number DQ380149) [27] were identical to each other.

### Viremia and clinical signs

**Figure 1:**
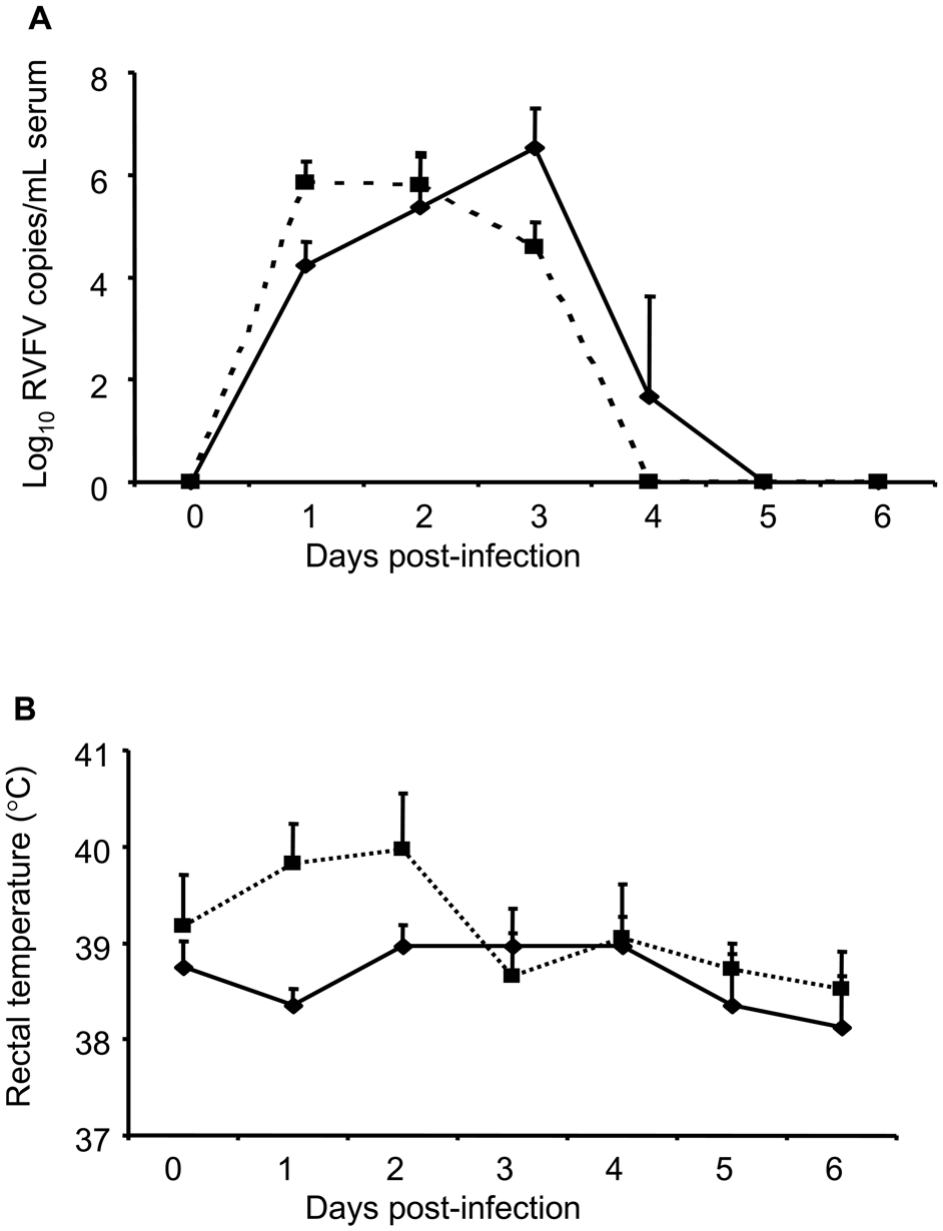
Viremia and rectal temperatures in Rift Valley fever virus (RVFV)-infected goats. A: Average viremia —▪— in IN-RVFV and —♦— MAM RVFV- infected goats (n = 4 goats each). B: Average rectal temperature for —▪— IN-RVFV and —♦— MAM RVFV- infected goats (n = 4 goats each). Data points represent means + standard deviation. The average rectal temperature of the goats in the 7 days prior to challenge was 38.9°C±0.5 standard deviation of mean. MAM-RVFV = RVFV produced in the mammalian cell line Vero E6, IN-RVFV = RVFV produced in the insect cell line C6/36.

All the goats infected with RVFV developed viremia starting at DPI 1. In IN-RVFV-infected goats, peak viremia was attained at DPI 1–2 and by DPI 4 all goats were aviremic (Figure 1A). On the other hand, MAM-RVFV-infected goats had peak viremia at DPI 3 and at DPI 4, 50% of the goats were still viremic (figure 1A). Indeed, on DPI 1 and 3 the difference in viremia between the 2 groups reached statistical significance (p<0.02). Peak viremia in MAM-RVFV-infected goats was higher than for IN-RVFV-infected goats but this difference was not statistically significant. By DPI 5, no virus could be detected in the blood of all the goats. The only clinical sign observed was a slight increase in rectal temperature in IN-RVFV infected goats. Following infection with IN-RVFV, rectal temperatures rose to 39.9–40.3°C, with maximum temperatures corresponding to peaks of viremia at DPI 1–2 (Figure 1B). In MAM-RVFV-infected goats, the increase in rectal temperature was barely noticeable, with a maximum of 39.9°C in 1 goat at DPI 2, and not exceeding 39.5°C in the rest of the goats (Figure 1B). However, there was no significant difference in rectal temperatures between the 2 groups. All goats survived through out the duration of the experiment.

### Changes in frequencies of cell types in PBMCs of RVFV-infected goats

**Figure 2:**
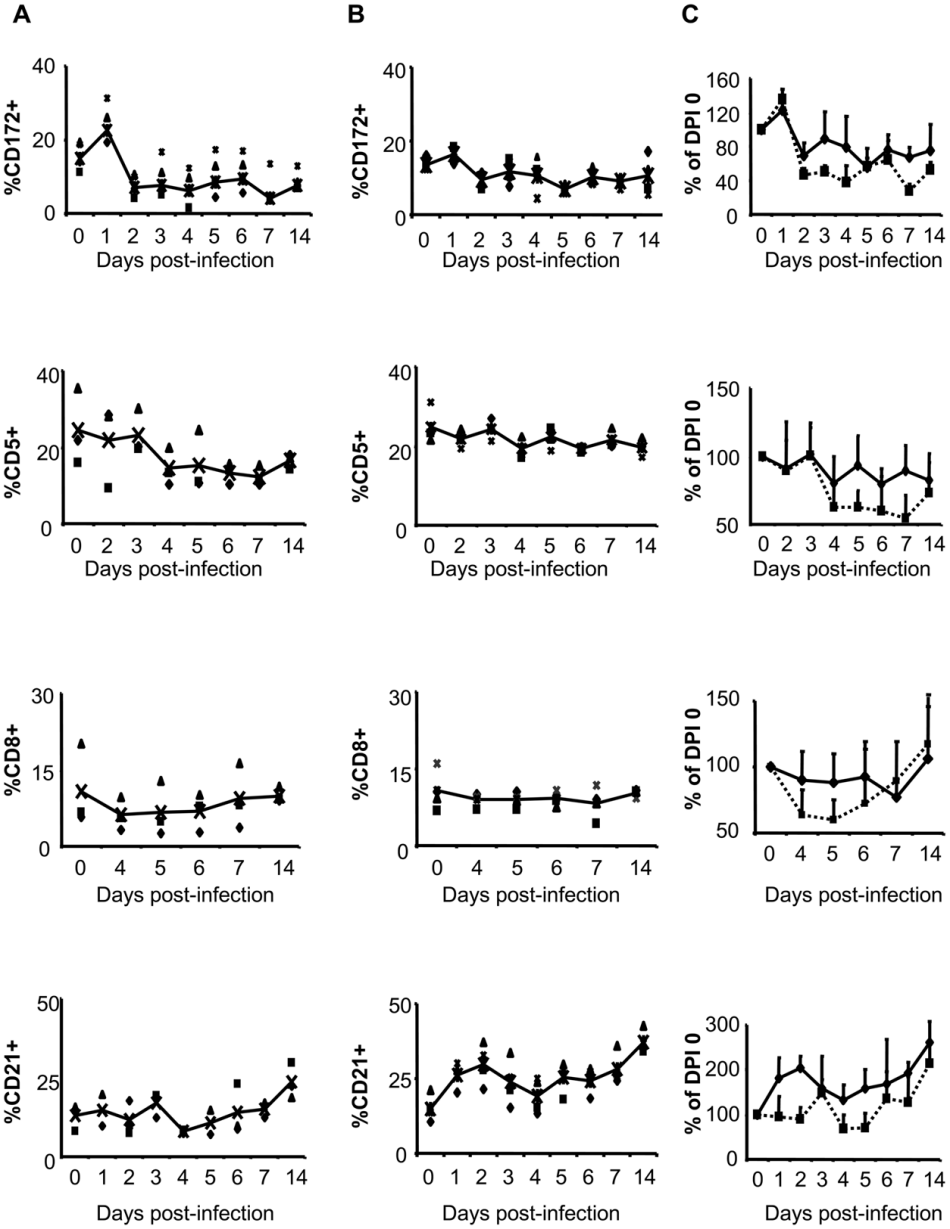
Changes in cell population frequencies in peripheral blood mononuclear cells in RVFV-infected goats. Column A: PBMCs from IN-RVFV-infected goats; Column B: PBMCs from MAM-RVFV-infected goats; Column C: Cell frequencies expressed as a percentage of pre-infection value for —▪— IN-RVFV and —♦—MAM RVFV- infected goats (n = 4 goats each). Data points in column A and B represent individual animals and the line represents the means. In column C data points represent means + standard deviation (error bars). MAM-RVFV = RVFV produced in the mammalian cell line Vero E6, IN-RVFV = RVFV produced in the insect cell line C6/36.

Monocyte/DC, T lymphocytes, cytotoxic T cells and B cells were identified with antibodies against CD172a, CD5, CD8 and CD21 surface markers respectively. Frequencies of these cells in naïve goats ranged from 11.1–19.3% (CD172a+ monocytes/DC), 15.8–35.4% (CD5+ T cells), 5.7–20.1% (CD8+ T cells) and 8.1–20.9% (CD21+ B cells). Following infection there was a drop in CD172a+ monocytes/DC starting on DPI 2 in both IN-RVFV and MAM-RVFV infected goats (Figure 2). However, this decline in CD172a+ monocytes/DC, expressed as a percentage of baseline frequencies, was more pronounced and statistically significant (p<0.01 on DPI 2 and 4, p<0.05 on DPI 3) in IN-RVFV-infected goats compared to MAM-RVFV-infected goats in which the drop never attained statistical significance (figure 2). On the other hand, CD5+ T cells and CD8+ T cells reduction was less than 20% in goats infected with MAM-RVFV while goats infected with IN-RVFV suffered approximately 40% drop (Figure 2). Conversely, in MAM-RVFV-infected goats, CD21+ B cell frequencies increased by approximately 2 fold on DPI 1 and 2 (p<0.05 and p<0.01 respectively), returning to within baseline values on DPI 3 but never dropping below baseline frequencies (Figure 2). On the contrary, in IN-RVFV-infected goats, the changes in CD21+ B cell frequencies were not statistically significant, only increasing slightly on DPI 3 but declining to 30% below baseline frequencies on DPI 4 and 5 (Figure 2). However, by DPI 14, CD21+ B cell frequencies were above baseline values in both groups.

### *Ex vivo* infectivity of goat PBMC by RVFV

**Figure 3:**
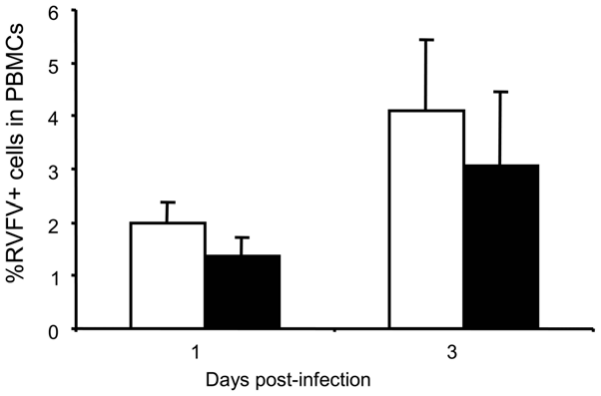
*Ex vivo* infection of peripheral blood mononuclear cells (PBMCs) in RVFV-infected goats. PBMCs isolated from goat blood at indicated time points post RVFV infection were stained for intracellular expression of RVFV non structural protein NSm1 and infected cells identified by flow cytometry. Means of RVFV+ PBMCs from IN-RVFV-infected goats are represented by the open histograms and MAM-RVFV infected goats by the filled histograms. Error bars represent standard deviation of means (n = 3 goats each).

To investigate whether the decline in frequencies of identified PBMC subsets was as a result of permissiveness to RVFV, PBMC from infected goats were examined for the presence of virus by intracellular staining for the non-structural protein (NSm1) and flow cytometry. At DPI 1, 1.4 to 2% of PBMCs in MAM-RVFV and IN-RVFV-infected goats were positive for RVFV which increased to 3 to 4% on DPI 3 (Figure 3). More PBMCs stained for NSm1 in IN-RVFV-infected than in MAM-RVFV-infected goats, reaching statistical significance at DPI 1 (p ≤ 0.05). However, this difference was not statistically significant at DPI 3.

### *In vitro* infectivity of goat MoDC by RVFV

**Figure 4:**
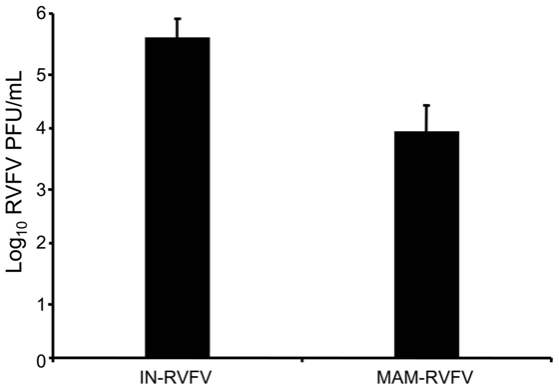
Virus yield from goat monocyte-derived dendritic cells (MoDCs) inoculated with RVFV. MoDCs were infected with insect cell-derived RVFV (IN-RVFV) or mammalian cell –derived RVFV (MAM-RVFV) and after 24 h, the virus in supernatants was quantified by plaque assay. Histograms represent means + standard deviation.

Since goat MoDCs have not been previously described, we first confirmed that the cells derived from goat monocytes with bovine GM-CSF and IL-4 had the phenotype of related bovine MoDCs [30], [35]. These cells had the morphology of DC and were CD14 negative, CD11c and CD172a low as opposed to MΦ that were CD14+, CD11c and CD172a high (supplementary figure 1). When these cells were infected with RVFV at MOI 0.1, approximately 1 log10 PFU/mL more virus (p<0.05) was obtained from IN-RVFV-infected MoDCs compared to MAM-RVFV at 24 h post infection (Figure 4).

### Serum cytokine response to RVFV infection in goats

**Figure 5:**
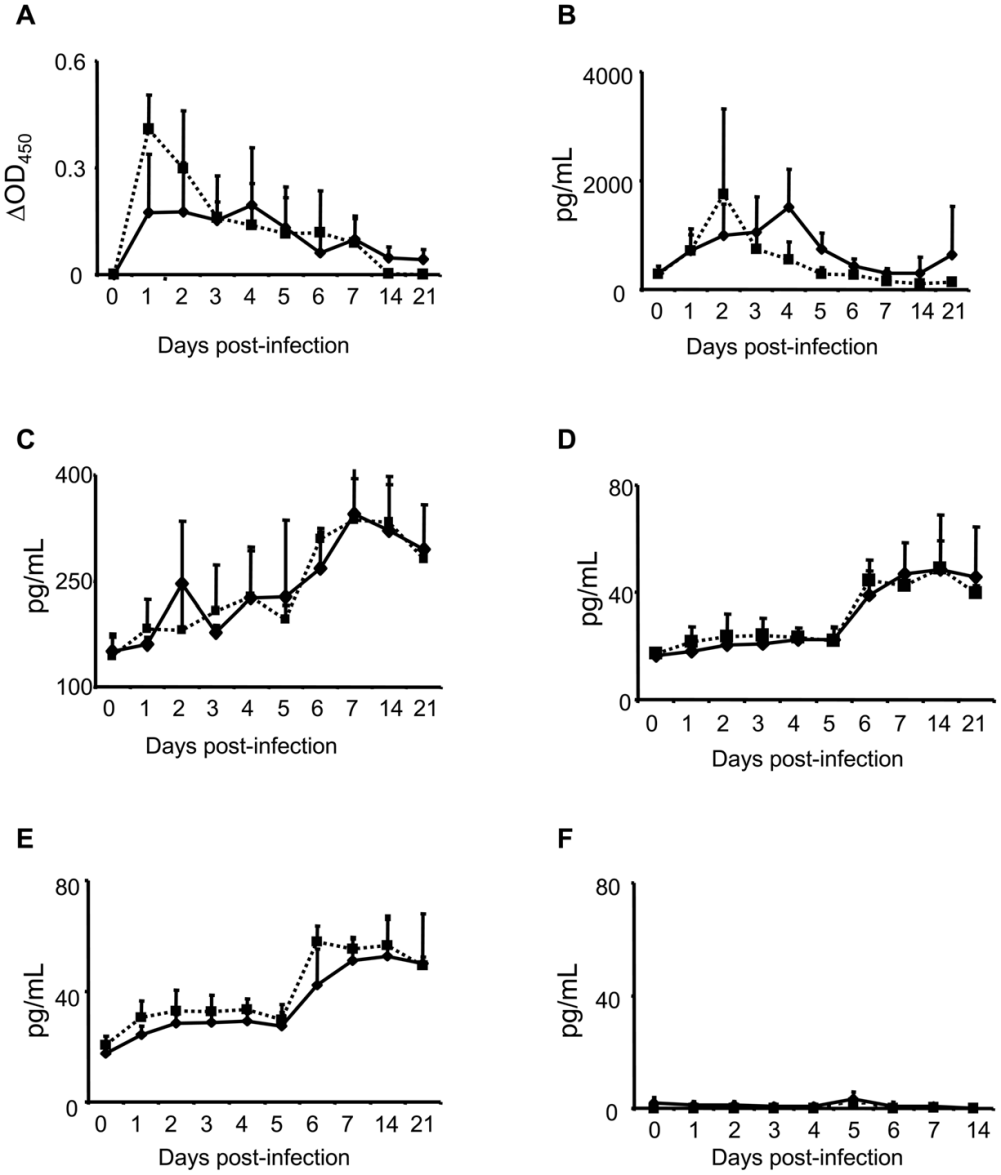
Serum cytokine response in RVFV-infected goats. A: Interleukin (IL)-12, B: interferon gamma, C: Tumour necrosis factor alpha, D: IL-6 and E: IL-1β and F: interferon alpha. —▪— IN-RVFV and —♦—MAM RVFV- infected goats (n = 4 goats each). Data points represent means + standard deviation. MAM-RVFV = RVFV produced in the mammalian cell line Vero E6, IN-RVFV = RVFV produced in the insect cell line C6/36.

RVFV infection in goats was characterized by 2 cytokine response patterns. Serum levels of IL-12 and IFN-γ peaked early post-infection while TNF-α, IL-6 and IL-1β levels peaked later. Serum IL-12 levels peaked at DPI 1 in both IN-RVFV-infected and MAM-RVFV-infected goats (Figure 5). The increase in IL-12 response for IN-RVFV-infected goats reached statistical significance on DPI 1 and 2 compared to baseline (p<0.01). On the contrary, the increase in IL-12 response for MAM-RVFV-infected goats did not reach statistical significance. The peak IL-12 response was significantly different between IN-RVFV-infected and MAM-RVFV-infected goats (p = 0.03). However, when all infected goats were analysed together, the IL-12 response was significant at DPI 1 (p<0.01) and DPI 2 (p<0.05). Maximum levels of serum IFN-γ was reached in IN-RVFV-infected goats at DPI 2 but this was delayed until DPI 4 in MAM-RVFV-infected goats (Figure 5). The IFN-γ response reached statistical significance on DPI 2 in IN-RVFV-infected goats (p<0.05) and DPI 4 in MAM-RVFV-infected goats (p<0.05) compared to baseline. There was no significant difference in peak IFN-γ response between the 2 groups. Serum TNF-α, IL-6 and IL-1β levels rose slightly at DPI 1 followed by a significant increase (p<0.05) at DPI 6 (Figure 5). There were no significant differences between IN-RVFV-infected and MAM-RVFV-infected goats with regards to TNF-α, IL-6 and IL-1β response. Minute levels of IFN-α (≤5 pg/ml) were detected in serum from some naïve goats but these levels did not increase early post infection (Figure 5).

### *In vitro* antiviral effect of interferon gamma against RVFV

Due to the high serum IFN-γ response in RVFV-infected goats, direct antiviral effect of this cytokine on RVFV was tested *in vitro* using recombinant bovine IFN-γ. IFN-γ had minimal effect against RVFV, with only 21% plaque inhibition at 1000 ng/mL (data not shown). In addition, this inhibition was dose dependent, with none observed at 8 ng/mL concentration.

### Adaptive cell-mediated immune response to RVFV

**Figure 6:**
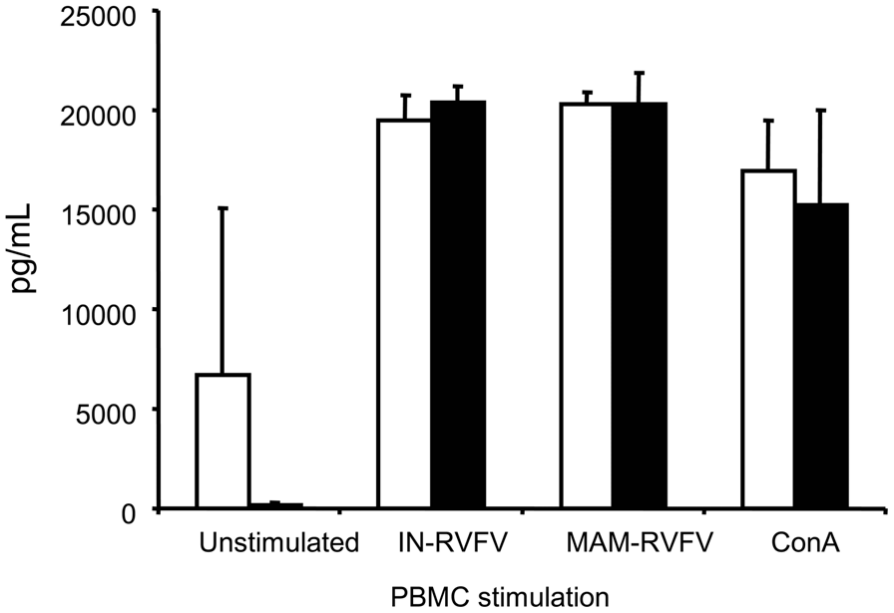
Adaptive cell mediated immunity in RVFV-infected goats. Means of PBMC IFN-γ response from IN-RVFV-infected goats are represented by the open histograms and MAM-RVFV infected goats by the filled histograms. Error bars represent standard deviation of means (n = 4 goats each). MAM-RVFV = RVFV produced in the mammalian cell line Vero E6, IN-RVFV = RVFV produced in the insect cell line C6/36.

*In vitro* RVFV-induced IFN-γ secretion by PBMCs from convalescent goats was used to determine specific cell-mediated immunity (CMI) [31]. PBMCs harvested from convalescent goats at DPI 21 secreted high levels of IFN-γ in response to RVFV re-exposure (Figure 6). This IFN-γ response was almost identical between the IN-RVFV-infected and MAM-RVFV-infected goats irrespective of which virus (IN-RVFV or MAM-RVFV) was used for *in vitro* re-stimulation of PBMCs. In addition, the response to the non-specific mitogen, ConA, was similar in both groups. Unstimulated cells secreted significantly less IFN-γ compared to the cells exposed to either virus (p<0.02).

### Antibody response in RVFV-infected goats

Antibody response, based on PRNT_70_, commenced on DPI 5 with low titres in most of the goats. Antibody titres rose to a maximum at DPI 21–30 in all goats. However, PRNT_70_ titres for IN-RVFV infected goats at DPI 7, 14 and 21 were significantly lower (p<0.05) compared to MAM-RVFV infected goats (Table 1). There was no significant difference in antibody response between the 2 groups at DPI 30.

**Table 1:** Antibody response in RVFV-infected goats.

		PRNT_70_ titre							
Group	Goat #	DPI 0	DPI 4	DPI 5	DPI 6	DPI 7	DPI 14	DPI 21	DPI 30
MAM-RVFV	4	<20	<20	20	40	40	320	640	640
	27	<20	<20	20	40	80	320	640	640
	28	<20	<20	20	20	80	320	1280	5120
	44	<20	<20	20	40	40	320	1280	640
IN-RVFV	29	<20	<20	20	20	20	80	320	320
	38	<20	<20	20	20	40	40	320	640
	39	<20	<20	20	20	20	40	320	640
	48	<20	<20	40	40	40	40	320	640

Antibody response to RVFV was measured by plaque reduction neutralisation test (PRNT). The reciprocal of the serum dilution giving at least 70% plaque inhibition relative to the virus control was taken as the PRNT_70_ titre for that sample. MAM-RVFV = mammalian cell-derived RVFV, IN-RVFV = insect cell-derived RVFV.

## Discussion

Cattle, sheep and goats have long been recognized as the natural hosts of RVFV. The clinical manifestation and pathology of natural and experimental RVF in cattle and sheep have been reported [8], [31], [36]–[38]. To the best of our knowledge, only one report of experimental infection in goats is published, and it did not address the innate immune response to the virus in goats [18]. Pathology following vaccination of goats with an attenuated strain of RVFV has been studied. However reports from other models reveal that this is not the same as infection with the virulent strain [19]. In this report we attempted to address some aspects of the innate and adaptive immune response to RVFV in goats. In addition, we compared these parameters between insect cell-derived and mammalian cell-derived RVFV. As in the previous report [18], the incubation period for RVFV in goats was 24 h. A similar incubation period is recorded for sheep, cattle, non-human primates and humans [3]. Peak viremia at DPI 1–3 was similarly reported in goats [18] and other susceptible species [3], [11], [31]. There were no mortalities and the only clinical sign we observed in these RVFV-infected goats was a mild fever in a subset of animals. Therefore, experimental infection of goats with RVFV produces a fairly typical disease course similar to what has been observed in other ruminants of a similar age group [3], [18], [31].

We observed a significant decline in CD172a+ cells (monocytes and dendritic cells) in RVFV-infected goats. There was also a pronounced decline in T cells (CD5+) and a transient decline in cytotoxic lymphocytes (CD8+) in IN-RVFV infected goats. Only a slight decline in the CD5 population was observed for MAM-RVFV infected goats. It has been suggested that RVFV can directly cause necrosis in infected cells as part of the disease pathogenesis [25], [39] and RVFV has been isolated from human PBMCs in a natural outbreak [40]. RVFV has also been shown to infect human monocytes/macrophages [25], [41]. Furthermore, RVFV was detected in Kupffer cells (resident liver macrophages) [39]. The differential effect of IN-RVFV and MAM-RVFV on PBMCs could be due to their differential ability to infect PBMC subsets. Indeed, PBMCs from IN-RVFV-infected goats had significantly higher percentage of RVFV NSm1 positive cells than in their MAM-RVFV-infected counterparts at DPI 1 which might be linked to the observation of a more profound decline in CD172a+, CD5+ and CD8+ cells in IN-RVFV infected goats. This is further supported by our *in vitro* data which shows that IN-RVFV infects MoDCs more readily than does MAM-RVFV. Furthermore, RVFV has previously been shown to infect MoDCs [21]. In addition, in arboviruses, insect cell-derived *alphaviruses* infect MoDCs more efficiently than mammalian cell-derived ones. The presence of high mannose carbohydrates in the viral glycoproteins is thought to enable the former to readily bind receptors on target cells [20], [22]. Contrary to the other cell subsets, CD21+ B cell frequencies increased post infection and never dropped below baseline in MAM-RVFV infected goats, while the slight increase in CD21+ B cell frequencies in IN-RVFV infected goats was followed by a decline below baseline frequencies. The amplification of B cells probably prepared the immune system for the more robust antibody production in MAM-RVFV infected goats as opposed to in IN-RVFV infected ones.

To the best of our knowledge, cytokine response to RVFV in ruminants has not been investigated. Here we report the detection of IL-12, IFN-γ, TNF-α, IL-6 and IL-1β in serum of RVFV-infected goats. Also of significance, is the absence of detectable IFN-α, one of the most potent antiviral cytokines. Experimental models have demonstrated a role for IFN-α in RVFV clearance [11] and the virus has developed mechanisms, via the NSs protein, to inhibit IFN-α response in infected cells [12]–[14], [25]. The presence of other cytokines but not IFN-α in RVFV-infected goats suggests that the virus may have specifically blocked its production/induction. This would create a window for high viremia to be attained which usually occurs within 24 h of infection. On the other hand, IL-12 and IFN-γ peaked at DPI 2–4 suggesting an otherwise functional innate immune response to RVFV in goats. In previous reports in sheep, RVFV was cleared from blood several days before the detection of neutralising antibodies indicating that innate immunity was likely responsible for this early protection [31]. IL-12 is known to activate bovine and ovine NK cells to secrete IFN-γ [42] which in turn activates NK cells to better cytotoxicity [43]. The response pattern in the current report suggests that IL-12 might have promoted the IFN-γ response, possibly from NK cells though other cells including macrophages and DC also secrete IFN-γ [44]. In previous studies [10], monkeys were protected from RVF when human IFN-γ was administered 24 h prior to infection. In addition to promoting NK cell cytotoxicity and downstream adaptive immune responses, IFN-γ is known to activate pathways that can directly inhibit virus [43]. However, using recombinant bovine IFN-γ, we did not detect any significant direct antiviral effect on RVFV replication in MDBK cells. Indeed, there was no antiviral effect at titres equivalent to the maximum serum IFN-γ response in RVFV-infected goats. Furthermore, it has been demonstrated that human IFN-γ has minimal *in vitro* antiviral effect against RVFV [45]. It is therefore, possible that IFN-γ and IL-12 may have played a role in the rapid clearance of viremia in RVFV-infected goats by activating NK cells, even though a direct antiviral effect of these cytokines can not be ruled out. This will be investigated in subsequent experiments. The other pro-inflammatory cytokines may have also played a role in RVFV clearance despite reaching peak levels on DPI 6–7. Recent data from humans suggests that a strong pro-inflammatory response is linked to survival of RVF [25].

The detection of neutralising antibodies starting at DPI 5 reported here has been similarly observed in natural and experimental infections in other animal models and humans [2], [15]–[17]. Neutralising antibodies are believed to be crucial for the early protection against RVFV [2]. Based on our observations in goats, the initial protection could be primarily due to innate immunity (mediated by cytokines and possibly NK cells). Nevertheless, neutralising antibodies are responsible for long term protection from subsequent challenge [2]. Adaptive cell mediated immunity may also be involved in long term protection from RVFV as suggested by the high IFN-γ response following restimulation of cells from convalescent goats (this report) and sheep [31]. Experimental trials in mice have also suggested that cell mediated immunity is important for post-vaccinal protection against RVFV [46].

In conclusion, experimental RVF in goats closely resembles natural and experimental infection in other ruminant hosts. Apparently, the virus infects DCs and monocytes and inhibits IFN-α response thereby allowing rapid replication. However other arms of innate and possibly adaptive immunity combine to protect animals from RVFV shortly after infection. The source of virus appears to influence events during infection, with IN-RVFV attaining peak viremia more rapidly, infecting more PBMCs, inducing slight fever and higher levels of early cytokines but lower levels of neutralising antibodies at onset of seroconversion. These findings seem to suggest that infections acquired from mosquito bites could differ somewhat from those due to contact with infectious material. However, this is far from conclusive considering the small sample size of 4 goats per group and the fact that in a natural setting things are much more complex, with other factors such as dose of infection, age and immune status likely to influence the course of disease. In addition, considering that these are outbred animals, genetic factors could also have contributed to the observed differences. Nevertheless, all 8 goats responded to RVFV by secreting cytokines irrespective of the source of virus. More work is required in goats and other ruminants to check if these results can be similarly observed in these species.

## Supporting Information

Figure S1 **Phenotype of goat monocyte-derived dendritic cells.** MoDCs were differentiated from adherent blood monocytes using recombinant bovine GM-CSF and IL-4. For controls, adherent monocytes were cultured in culture medium only (MΦ). Pictures were taken on days 0, 3 and 6. On day 7, MoDCs and MΦ were harvested and analyzed by flow cytometry. 1A. shows progression from monocytes to MoDCs with characteristic dendrites. 1B. shows MoDCs (broken line) as CD14 negative, CD172a and CD11c low as opposed to MΦ (solid line) that were CD14+, CD172a and CD11c high. Filled histograms represent cells stained with isotype control antibody.
(TIF)
