# Supplementary figures and images for: Innate Immune Response to Rift Valley Fever Virus in Goats
Source: PLoS Negl Trop Dis. 2012 Apr 24;6(4):e1623. doi: 10.1371/journal.pntd.0001623 (PMC3335883; doi:10.1371/journal.pntd.0001623)

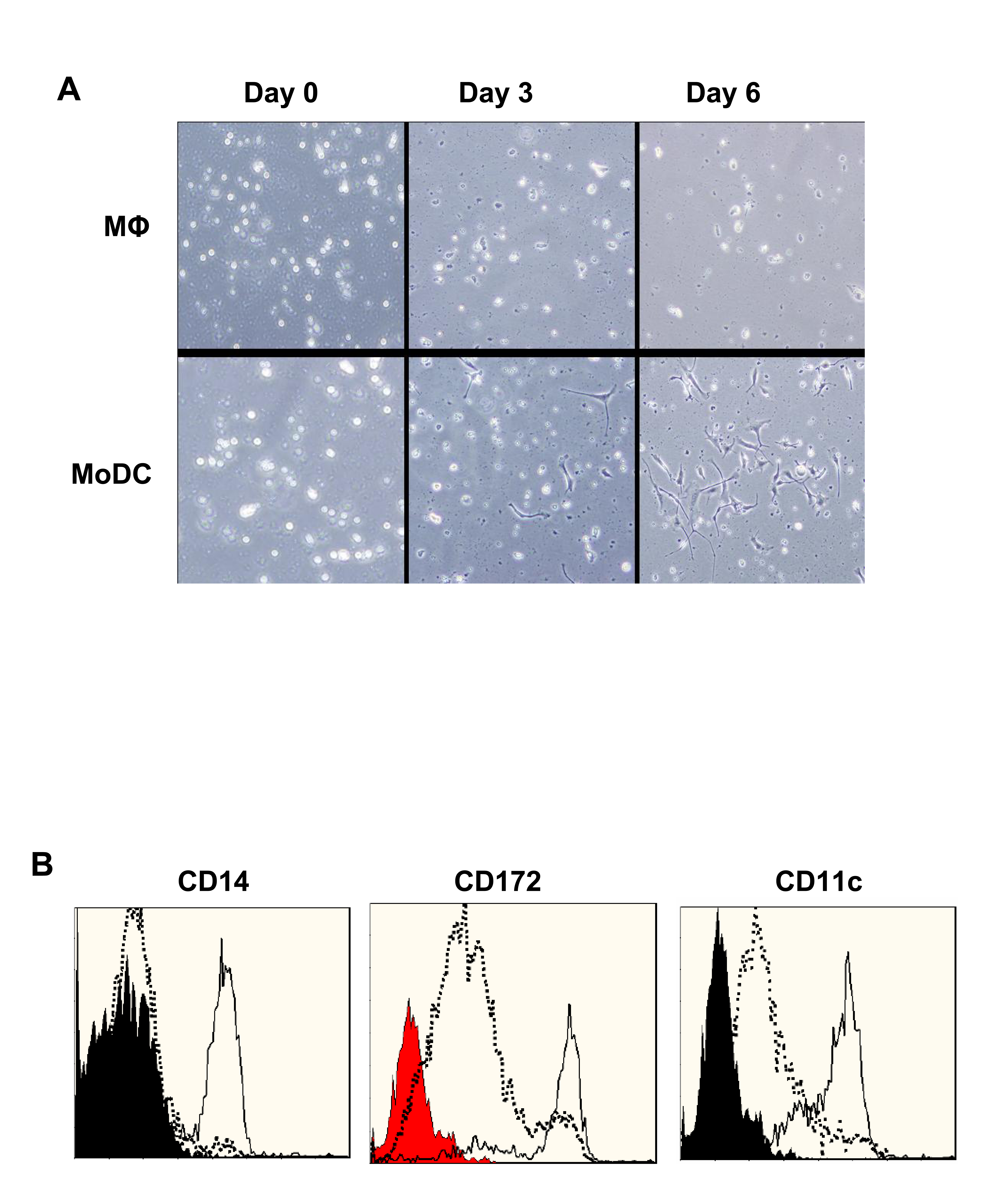

Supplement: Figure S1 — Phenotype of goat monocyte-derived dendritic cells. MoDCs were differentiated from adherent blood monocytes using recombinant bovine GM-CSF and IL-4. For controls, adherent monocytes were cultured in culture medium only (MΦ). Pictures were taken on days 0, 3 and 6. On day 7, MoDCs and MΦ were harvested and analyzed by flow cytometry. 1A. shows progression from monocytes to MoDCs with characteristic dendrites. 1B. shows MoDCs (broken line) as CD14 negative, CD172a and CD11c low as opposed to MΦ (solid line) that were CD14+, CD172a and CD11c high. Filled histograms represent cells stained with isotype control antibody. (TIF) [file pntd.0001623.s001.tif]
